# Tranexamic acid by the intramuscular or intravenous route for the prevention of postpartum haemorrhage in women at increased risk: a randomised placebo-controlled trial (I’M WOMAN)

**DOI:** 10.1186/s13063-023-07687-1

**Published:** 2023-12-03

**Authors:** Amy Brenner, Haleema Shakur-Still, Rizwana Chaudhri, Projestine Muganyizi, Oladapo Olayemi, Monica Arribas, Aasia Kayani, Kiran Javid, Adenike Bello, Ian Roberts, on behalf of the I’M WOMAN Trial Collaborative Group

**Affiliations:** 1 London School of Hygiene & Tropical Medicine, London, United Kingdom; 2 Shifa Tameer-E-Millat University, Islamabad, Pakistan; 3 University of Dar Es Salaam, Dar Es Salaam, Tanzania; 4 University of Ibadan, Ibadan, Oyo Nigeria

**Keywords:** Antifibrinolytic, Tranexamic acid, Postpartum haemorrhage, Clinical trial, Intramuscular, Intravenous, Route of administration

## Abstract

**Background:**

Postpartum haemorrhage (PPH) causes about 70,000 maternal deaths every year. Tranexamic acid (TXA) is a life-saving treatment for women with PPH. Intravenous (IV) TXA reduces deaths due to PPH by one-third when given within 3 h of childbirth. Because TXA is more effective when given early and PPH usually occurs soon after childbirth, giving TXA just before childbirth might prevent PPH. Although several randomised trials have examined TXA for PPH prevention, the results are inconclusive. Because PPH only affects a small proportion of births, we need good evidence on the balance of benefits and harms before using TXA to prevent PPH. TXA is usually given by slow IV injection. However, recent research shows that TXA is well tolerated and rapidly absorbed after intramuscular (IM) injection, achieving therapeutic blood levels within minutes of injection.

**Methods:**

The I’M WOMAN trial is an international, multicentre, three-arm, randomised, double-blind, placebo-controlled trial to assess the effects of IM and IV TXA for the prevention of PPH in women with one or more risk factors for PPH giving birth vaginally or by caesarean section.

**Discussion:**

The trial will provide evidence of the benefits and harms of TXA for PPH prevention and the effects of the IM and IV routes of administration. The IM route should be as effective as the IV route for preventing bleeding. There may be fewer side effects with IM TXA because peak blood concentrations are lower than with the IV route. IM TXA also has practical advantages as it is quicker and simpler to administer. By avoiding the need for IV line insertion and a slow IV injection, IM administration would free up overstretched midwives and doctors to focus on looking after the mother and baby and expand access to timely TXA treatment.

**Trial registration:**

ClinicalTrials.gov NCT05562609. Registered on 3 October 2022. ISRCTN Registry ISRCTN12590098. Registered on 20 January 2023. Pan African Clinical Trial Registry PACTR202305473136570. Registered on 18 May 2023.

**Supplementary Information:**

The online version contains supplementary material available at 10.1186/s13063-023-07687-1.

## Administrative information

Note: The numbers in curly brackets in this protocol refer to the SPIRIT checklist item numbers. The order of the items has been modified to group similar items (see http://www.equator-network.org/reporting-guidelines/spirit-2013-statement-defining-standard-protocol-items-for-clinical-trials/).

**Table:** 

Title {1}	Tranexamic acid by the intramuscular or intravenous route for the prevention of postpartum haemorrhage in women at increased risk: a randomised placebo-controlled trial (I’M WOMAN)
Trial registration {2a and 2b}	ISRCTN, ISRCTN12590098, Registered 20th January 2023, https://doi.org/10.1186/ISRCTN12590098.
Protocol version {3}	Version 2.0, dated 1 June 2023
Funding {4}	The project is funded by Unitaid.
Author details {5a}	I’M WOMAN Trial Collaborative Group
Name and contact information for the trial sponsor {5b}	Research Governance & Integrity Office London School of Hygiene & Tropical Medicine, Keppel Street London, WC1E 7HT, UK Phone: + 44 (0)20 7927 2626 Email: RGIO@lshtm.ac.uk
Role of sponsor {5c}	The sponsor and funder have no role in the design; collection, management, analysis and interpretation of the data; writing of the report; or the decision to submit the report for publication, nor do they will ultimate authority over any of these activities.

## Introduction

### Background and rationale {6a}

Postpartum haemorrhage (PPH) is a leading cause of maternal death, responsible for about 70,000 deaths each year, worldwide [1, 2]. Tranexamic acid is a life-saving treatment for women with PPH. The WOMAN trial recruited over 20,000 women with PPH and found that intravenous (IV) tranexamic acid given soon after PPH onset reduces bleeding deaths by about one-third [3]. The World Health Organization (WHO) recommends that all women with PPH should receive tranexamic acid as a first-line treatment [4].

Tranexamic acid is more effective when given early, around the time bleeding starts. Every 15-min delay reduces the survival benefit by about 10% [5]. PPH usually occurs soon after childbirth. This suggests that giving tranexamic acid around the time of childbirth might prevent PPH. Although several clinical trials have examined the effectiveness of tranexamic acid for the prevention of PPH, the results are inconclusive [6–8]. Some bleeding-related outcomes were reduced by tranexamic acid but others were not, and in some trials, rates of nausea and vomiting were increased with intravenous tranexamic acid. Trials of tranexamic acid for PPH prevention give the trial treatment after cord clamping, which may be too late to prevent heavy bleeding in some women, as bleeding usually happens soon after childbirth [6–9].

Before using tranexamic acid to prevent PPH, we need good evidence of the benefits and any potential harms. Only a small proportion of births are complicated by PPH, and even when PPH does occur, most women survive. Assuming the risk of PPH is 5% and that tranexamic acid cuts this risk by 20%, we would need to treat 100 women to prevent one PPH. If there are no important harms, this might be acceptable, but if there are harms, the balance of benefits and harms will need careful consideration.

The ongoing WOMAN-2 trial is evaluating the effects of tranexamic acid at cord clamping for the prevention of PPH in women with moderate and severe anaemia having a vaginal birth [9]. Anaemic women have a much higher risk of PPH and are more likely to die from bleeding if PPH occurs [10–12]. If tranexamic acid is found to be effective, the balance of benefits and harms should be favourable in anaemic women.

The I’M WOMAN trial will evaluate the effects of tranexamic acid just before birth for the prevention of PPH in women with one or more risk factors for PPH giving birth vaginally or by caesarean section (CS). The results will deepen our understanding of the benefits and harms of tranexamic acid for PPH prevention. The trial will also evaluate the effect of the route of tranexamic acid administration. Tranexamic acid is usually given by slow IV injection. However, recent research shows that tranexamic acid is well tolerated and rapidly absorbed after intramuscular (IM) injection, achieving therapeutic blood concentrations within minutes of injection [13–17]. The intramuscular route should be as effective as the IV route for preventing bleeding.

There may be fewer side effects with IM tranexamic acid. In general, the risk of side effects is increased at high blood concentrations. Although tranexamic acid is rapidly absorbed after IM injection, reaching therapeutic concentrations within minutes, because of the absorption phase, peak blood concentrations are lower than with the IV route. By avoiding high blood concentrations, the IM route might cause fewer side effects (e.g. nausea, vomiting, dizziness). A better safety profile would be particularly important when using tranexamic acid for PPH prevention. The IM route would also have practical advantages. By avoiding the need for IV line insertion and a slow IV injection, it would free up overstretched midwives and doctors to focus on looking after the mother and baby.

Giving tranexamic acid before the bleeding becomes serious should maximise the benefits [5]. In vaginal births, bleeding can start after episiotomy or birth canal trauma as the baby is being born, so giving tranexamic acid at crowning should be more effective at preventing bleeding. In caesarean births, bleeding starts at the skin incision. Women having a CS have an increased risk of PPH, surgical blood loss can be substantial and there is strong evidence that preoperative tranexamic acid safely reduces surgical bleeding [18, 19]. Giving tranexamic acid just prior to skin incision should be most effective. A safety study of IV, IM and oral tranexamic acid in pregnant women having caesarean births found no evidence of maternal or neonatal adverse events. Tranexamic acid crosses the placenta but has a short half-life and is rapidly eliminated [20].

### Objectives {7}

The I’M WOMAN trial is a randomised, placebo-controlled trial to assess the effects of IM and IV tranexamic acid on PPH, adverse events and other important maternal health outcomes in women at increased risk of PPH. It will:

1. Assess the effect of tranexamic acid on the risk of PPH and other bleeding-related outcomes
2. Compare the effects of IM and IV tranexamic acid on the risk of PPH
3. Compare the effects of IM and IV tranexamic acid on the risk of adverse events

### Trial design {8}

The I’M WOMAN trial is a parallel, randomised, double-blind, placebo-controlled, three-arm trial comparing the effects of tranexamic acid by the intramuscular and intravenous route with placebo in women with one or more risk factors for PPH giving birth vaginally or by CS. About 30,000 women will be allocated to receive either (a) 1 g of tranexamic acid as two 5-ml IM injections (100 mg/ml) and IV placebo (10 ml 0.9% sodium chloride), (b) 1 g of tranexamic acid by IV injection and two 5-ml IM placebo injections, or (c) matching placebo. An optimal, allocation ratio was used to maximise study power.

## Methods: participants, interventions, and outcomes

### Study setting {9}

We will conduct the trial in hospitals in Africa and South Asia where maternal mortality from PPH is high. Study sites will be listed in country-specific protocols and on the trial website (https://imwoman.lshtm.ac.uk/). Participating investigators and sites will be identified from the international network of health professionals that was established for the previous WOMAN trials. Before we start the trial, all relevant ethics and regulatory approvals will be in place. All principal investigators will be required to conduct the trial according to the protocol, Good Clinical Practice (GCP) guidelines, and other relevant regulations. We will only progress countries and sites able to obtain the necessary ethics and regulatory approvals in acceptable timeframes.

### Eligibility criteria {10}

For a woman to be eligible, the randomising clinician must be uncertain about whether to give tranexamic acid. If the clinician believes tranexamic acid is indicated based on the existing evidence, they should not randomise the woman into the trial. During the trial, if accumulating evidence from other trials clearly demonstrates that tranexamic acid prevents PPH with a favourable balance of benefits and harms, it would be unethical to randomise women to receive a placebo. If this happens, the placebo arm will be dropped, and we will continue to recruit women into the IM and IV TXA arms.

#### Inclusion criteria

Women thought to be aged 18 years or older admitted to hospital for a vaginal or caesarean birth and who are known to have one or more risk factors for PPH will be potentially eligible. A list of major risk factors is provided in Additional file 8. If accumulating trial data or newly published research suggests that a particular factor is not strongly associated with an increased risk of PPH, we will focus on recruiting women with other/additional risk factors.

#### Exclusion criteria

The fundamental eligibility criterion is the responsible doctor’s ‘uncertainty’ about whether to use tranexamic acid in a particular woman. Women should not be randomised if the responsible doctor believes that tranexamic acid is clearly indicated (e.g. you have given tranexamic acid within 12 h or plan to give tranexamic acid) or clearly contraindicated (e.g. known allergy to tranexamic acid).

Site eligibility will be assessed by collecting information on the experience and qualifications of the site staff, the number and route of births, and the incidence of PPH.

### Who will take informed consent? {26a}

If women can give fully informed consent, then information about the trial will be given and written consent will be obtained. An overview of the consent procedure is provided in Additional file 2.

First, a member of the site trial team will identify a potentially eligible woman and then approach the woman with the agreement of the primary carer. She will be given information about the trial (Additional file 4a) in a language she understands. The team member will explain the purpose of the trial that it does not involve any change to her birth plan and that she will receive all the usual interventions for preventing PPH and any other care she needs. The team member will explain that her participation is voluntary and that if she does not want to take part, we will respect her views and her decision will not affect her care. If she wants to take part, the team member will obtain written consent (Additional file 4). If she is unable to read or write, the participant information sheet will be read to her, and she will mark the consent form with a cross or thumbprint. In this case, an impartial witness must provide a signature confirming the mark. A copy of the information sheet and consent form will be given to the woman.

If the woman withdraws a previously given informed consent, data collected to the point of withdrawal of consent will be used as part of the analysis.

### Additional consent provisions for collection and use of participant data and biological specimens {26b}

N/A—no biological samples will be collected. Consent is obtained for the study staff to access participants’ medical records and for their anonymised data to be made freely available to the public.

### Interventions

#### Explanation for the choice of comparators {6b}

The I’M WOMAN trial has an active control (IV tranexamic acid) and a placebo control group. A three-arm trial with experimental treatment, active control and placebo group is considered the gold standard non-inferiority design by drug regulatory agencies and is strongly recommended by the International Council for Harmonisation of Technical Requirements for Pharmaceuticals for Human Use (ICH) and European Medicines Agency (EMA). The IM and IV tranexamic acid groups will be compared to assess non-inferiority. The placebo group will be compared to the tranexamic acid groups to confirm assay sensitivity and provide evidence about the effects of tranexamic acid on PPH prevention. While tranexamic acid is recommended for the treatment of PPH, it is not recommended for prevention, and the balance of benefits and risks in women giving birth remains uncertain based on existing evidence, so a placebo arm is ethically and scientifically justified.

#### Intervention description {11a}

##### Name and description of investigational medicinal product

Tranexamic acid is a synthetic derivative of the amino acid lysine that has an antifibrinolytic effect by blocking lysine binding sites on plasminogen, inhibiting the binding of plasminogen to fibrin [21]. It is sold under various trade names for the treatment of bleeding due to general or local fibrinolysis in adults and children from 1 year of age. It is a well-known drug with an excellent safety profile.

##### Drug administration and dosage schedule

Women will be randomly allocated to receive:

i) 1 g tranexamic acid as 2 × 5 ml IM injections (100 mg/ml) and slow IV injection of placebo (1 × 10 ml of 0.9% sodium chloride)
ii) 1 g tranexamic acid by slow IV injection (1 × 10 ml) and 2 × 5 ml IM injections of placebo
iii) Placebo by 1 × slow 10-ml IV injection and 2 × 5 ml IM injections

In caesarean births, the trial treatment will be given prior to skin incision, immediately after draping. In vaginal births, the trial treatment will be given at crowning. There should be no delay in administering the trial treatment. Both IM injections should be given first, then the IV injection.

For IM administration, the 1 g dose (10 ml) is divided into two 5-ml IM injections to reduce the injection volume (5 ml is considered the upper limit) [22]. The IM injections should be given into the vastus lateralis, ventrogluteal region or deltoid. The vastus lateralis is the preferred site because it is a large muscle with no major nerve structures [23].

Each treatment pack contains clearly labelled IM and IV treatments. The IM treatment kit has two ampoules each containing 500 mg (5 ml) of TXA or placebo and two syringe labels. Two sterile 5-ml syringes and two needles will be provided. The IV treatment kit has two ampoules each containing 500 mg (5 ml) of TXA or placebo and one syringe label. One sterile 10-ml syringe and one needle will be provided. Before administration of IM and IV treatments, the expiry dates should be checked and the randomisation number confirmed. Appropriately qualified staff will prepare and administer the IM treatment first then the IV treatment:

IM treatment: Open the IM treatment kit and draw up the contents of each ampoule into each 5-ml syringe using the needles provided. Administer the two 5-ml IM injections first.

IV treatment: Open the IV treatment kit and draw up the contents of both ampoules into one 10-ml syringe using the needle provided. Administer one 10-ml injection as a slow intravenous injection at a rate of about 1 ml/min using the usual IV administration procedure, after administering the two IM injections.

##### Benefits and harms

Early tranexamic acid treatment cuts the risk of bleeding deaths in PPH and trauma by a third [3, 24, 25]. Giving tranexamic acid just before surgery cuts blood loss and the need for a blood transfusion by about a third to one-quarter [19, 26]. Although women in the postpartum period have an increased risk of venous thrombosis [27], meta-analyses of randomised trials with tens of thousands of patients show no increase in thromboembolic events with tranexamic acid, even in women with PPH[3, 18, 24, 28, 29]. Because severe bleeding is a strong risk factor for thromboembolic events, tranexamic acid might even reduce the risk of thrombosis [30]. The risk of seizures is increased at high doses but not with the dosage used in this trial [29]. Tranexamic acid is mostly excreted within 12 h, and there should be no risk of accumulation with a single dose. Unpublished data indicates that tranexamic acid passes into breast milk at about 1/100 of the concentration in the maternal blood, so this is very unlikely to produce an effect in the infant [31]. There was no increase in adverse events in the infants of mothers who received tranexamic acid in the WOMAN trial. A randomised trial of IV, IM and oral tranexamic acid in 120 pregnant women found no serious adverse events in infant(s) despite some placental transfer [17].

Tranexamic acid is widely used and well tolerated. Potential adverse events reported by manufacturers to be associated with the use of tranexamic acid according to frequency are:

- Common (≥ 1/100 to < 1/10): diarrhoea, vomiting and nausea
- Uncommon (≥ 1/1000 to < 1/100): dermatitis allergic
- Rare: hypersensitivity reactions including anaphylaxis, convulsions, visual disturbances including impaired colour vision, malaise with hypotension (generally following a too fast intravenous injection) and arterial or venous thrombosis

##### Investigator’s Brochure (IB)

Information about tranexamic acid will be detailed in an Investigator’s Brochure (IB). The IB should be reviewed annually. Studies that provide reliable information on the safety and efficacy of TXA that would help investigators assess the benefits and harms of TXA use will be included. Additionally, relevant information on updates from manufacturers of TXA will be included.

##### Preparation and labelling of medication to be used in the trial

Tranexamic acid has marketing authorisation in the UK and will be purchased from the open market. Marketing authorisation guarantees that drug manufacture and release comply with Good Manufacturing Practice (GMP). A GMP-certified manufacturer will prepare the matching placebo (sodium chloride 0.9%). Tranexamic acid and placebo ampoules and packaging will be identical. A clinical trial supplies company will conduct the blinding process and first-stage qualified person (QP) release. The blinding process involves replacing the manufacturer’s label with the clinical trial label. Other than the randomisation number (used for pack identification) and route of administration, the label text will be identical on all ampoules and comply with clinical trial requirements. To check the blinding, known tranexamic acid will be compared with blinded samples from a random set of treatment packs to determine which are tranexamic acid. The samples will then be un-blinded to confirm the accuracy of the labelling.

##### Drug storage and supply

When a site is ready to start, a box of treatment packs will be sent by the LSHTM CTU Global Health Trials Group or the in-country coordinating centre. Site stock level depends on the site’s average recruitment rate. Each time a participant is randomised and entered into the trial database, one pack from the site’s stock will be automatically deducted. When the stock reaches the site’s minimum level, the LSHTM CTU Global Health Trials Group or in-country coordinating centre will send another box (or boxes). Sites should send screening and entry data to the LSHTM CTU Global Health Trials Group as soon as possible after randomisation (ideally within 24 h). Sites must report all used, lost or damaged trial treatment packs to the LSHTM CTU Global Health Trials Group on a Drug Accountability Log.

At each site, the treatment packs will be stored securely in a place where they are always accessible to the trial team for randomisation. Although tranexamic acid is heat stable, it will be stored in a dry place where it is protected from excessive heat and freezing. The expiry date of the trial treatment will be printed on the ampoule label, the treatment pack and the drug box. When a batch of treatment packs is close to expiry, the principal investigator (PI)/trial pharmacist/delegate will be asked to arrange the destruction of affected packs and record this on a Drug Destruction Form (DDF). When a site is to be closed, the PI/trial pharmacist/delegate will arrange the destruction of all unused packs and return a completed DDF to the LSHTM CTU Global Health Trials Group to confirm disposal.

#### Criteria for discontinuing or modifying allocated interventions {11b}

If any contraindication to the trial treatment develops after randomisation, the trial treatment should be stopped.

#### Strategies to improve adherence to interventions {11c}

Site investigators should record the date and time of trial treatment administration. If the trial treatment is not given, or is given outside of the prescribed period, a reason should be given. Adherence to the protocol will be monitored, and we will record whether the full dose was given.

#### Relevant concomitant care permitted or prohibited during the trial {11d}

All women should receive the usual care in labour and after birth. There is no restriction on the use of concomitant medication. Participation will not result in any needed treatment being withheld. Women who develop PPH should be treated in the usual way at their hospital, which may include tranexamic acid.

#### Provisions for post-trial care {30}

Where a woman returns to the hospital for any adverse event associated with the trial, her travel costs will be reimbursed. LSHTM accepts the responsibility attached to its sponsorship of the trial and, as such, would be responsible for claims for any non-negligent harm suffered by anyone because of participation in this trial. The indemnity is renewed on an annual basis and LSHTM assures that it will continue renewal of the indemnity for the duration of this trial.

### Outcomes {12}

#### Primary outcome

The primary outcome is a clinical diagnosis of primary PPH. This may be an estimated blood loss of more than 500 ml in vaginal birth or more than 1000 ml in caesarean birth or any blood loss sufficient to compromise haemodynamic stability within 24 h of birth. Haemodynamic instability is based on clinical judgement and assessed using clinical signs (low systolic blood pressure, tachycardia, reduced urine output). The total estimated blood loss at the time of PPH diagnosis and presumed cause(s) of PPH will be recorded.

The true event rate of the primary outcome in the trial population is unknown. Interim analyses may indicate a lower-than-expected event rate, or new information may emerge that alters assumptions about the treatment effect, affecting the power of the study. To ensure the study has adequate power, prior to unblinding we will allow the primary outcome to be changed, with any changes set out in a statistical analysis plan.

#### Secondary outcomes

Specific to caesarean births:

1. Intraoperative blood loss measured by quantifying the amount of blood in sponges and drapes used in surgery and blood loss from suctioning, excluding amniotic fluid
2. Surgery duration
3. Intraoperative whole blood/red cell transfusion
4. Postoperative haemoglobin (Hb) or packed cell volume (PCV) up to the end of the second postoperative day, if known

All births:

1. Postpartum blood loss measured using a calibrated obstetric drape starting immediately after vaginal birth or once the women are shifted to a bed in the observation area after CS surgery, for 1 h, or up to 2 h if bleeding continues after 1 h and the woman remains in bed
2. Blood pressure and heart rate (lowest recorded blood pressure reading and associated heart rate) up to 24 h after birth
3. Interventions for bleeding (uterotonics, non-trial TXA, blood transfusion, surgical and non-surgical interventions) within 24 h of birth
4. Nausea, vomiting and dizziness (when the calibrated drape is removed, the woman will be asked about her nausea, vomiting and dizziness during and since the birth) measured using the number of episodes and/or a score from 1 to 10
5. Pain or adverse skin reactions at injection sites (when the calibrated drape is removed, each injection site will be inspected for local reactions and the woman will be asked about pain at her injection sites)
6. Prespecified maternal adverse events up to discharge, death or 42 days postpartum (thromboembolic events, seizure, sepsis, organ dysfunction, coagulopathy)
7. Other maternal adverse events up to discharge, death or 42 days postpartum
8. Maternal mortality up to discharge, death or 42 days postpartum (all-cause, cause-specific, narrative)
9. Length of hospital stay
10. Days in intensive care unit (ICU)/high dependency unit (HDU)
11. Transfer to another hospital
12. Prespecified neonatal outcomes up to discharge, death or 42 days (breastfeeding, intracranial haemorrhage, pulmonary haemorrhage, bruising, thromboembolic event, seizure, stillbirth/intrapartum death, neonatal death, cause of death, congenital and genetic abnormalities, adverse events)

### Participant timeline {13}

**Fig. 1:**
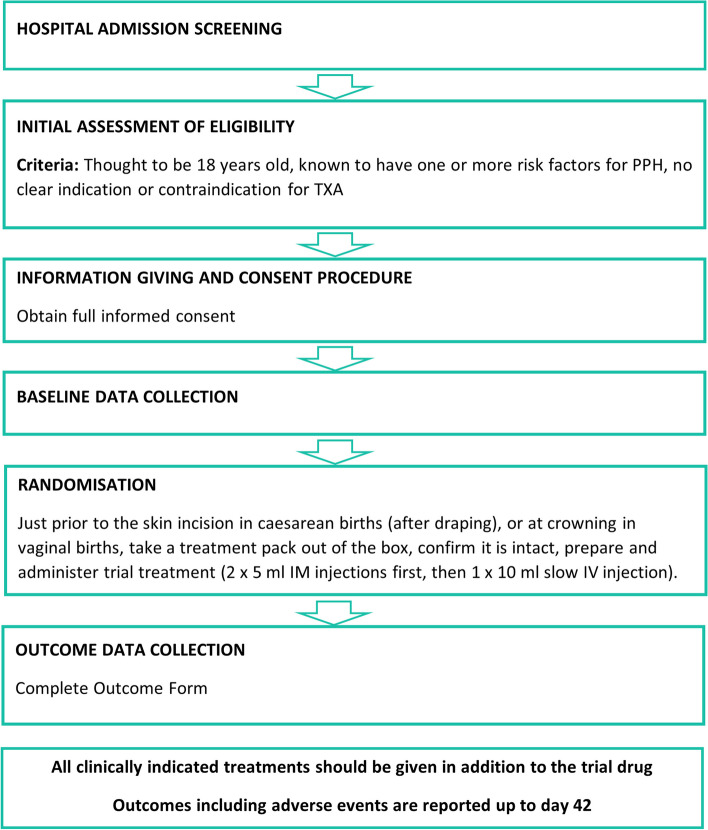
Participant timeline

**Fig. 2:**
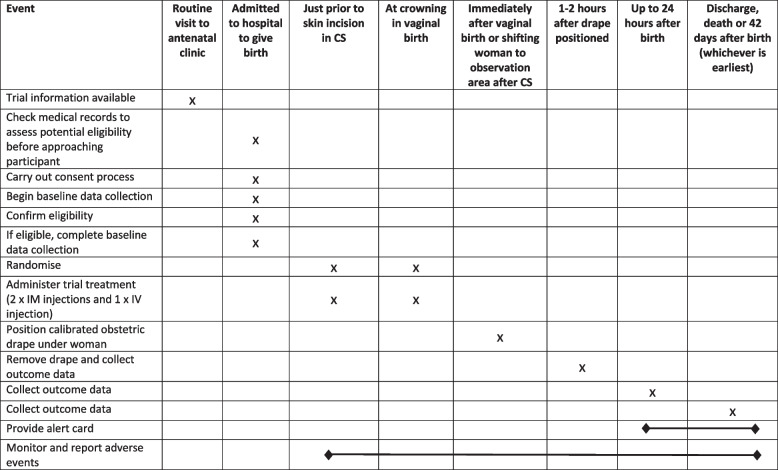
Schedule of events

Participant timeline and schedule of events are presented in Figs. 1 and 2.

### Sample size {14}

Two main factors determine the number of patients needed in a trial—the estimated event rate and the size of the treatment effect.

#### Estimated event rate

In trials of tranexamic acid for PPH prevention, the event rate (a clinical diagnosis of PPH) varies from 8 to 15% depending on the mode of delivery [6, 7, 9]. We assumed an event rate of 11.5% in the placebo group, based on a roughly 50:50 ratio of vaginal to caesarean births.

#### Size of the treatment effect

Based on the existing evidence in PPH prevention and surgical trials, we assumed a 25% reduction in PPH with tranexamic acid (RR = 0.75).

A trial of about 30,000 women has almost 90% power to detect that (1) tranexamic acid is more effective than placebo and (2) IM tranexamic acid is at least 60% as effective as IV tranexamic acid. We assumed a two-sided type 1 error rate of 5% for the superiority arm and a one-sided rate of 2.5% for the non-inferiority arm. Our sample size and power calculations were made using methods described by Stucke and Kieser (2012) [32]. We confirmed our results using simulation. An optimal allocation ratio was used to maximise study power with patients allocated to receive IM tranexamic acid, IV tranexamic acid and placebo.

Because the true event rate and treatment effect are unknown, we will re-estimate the sample size if unplanned interim analyses indicate a lower-than-expected event rate or if new information emerges which changes beliefs about the expected treatment effect.

### Recruitment {15}

We will use wall posters and brief information leaflets to inform pregnant women attending antenatal clinics and labour wards about the trial (see Additional file 3). Information may also be provided in videos. To avoid unnecessarily approaching women who will not be suitable, the site investigator will screen the medical records of women attending the hospital to give birth to see if they have a risk factor(s) for PPH and check how old they are or are thought to be. The site investigator will approach eligible women and invite them to take part in the trial and carry out formal screening. This will first involve giving information and seeking consent.

### Assignment of interventions: allocation

#### Sequence generation {16a}

An IT coding expert supported by a statistician will prepare a randomisation list detailing the allocation sequence (the order in which treatment groups are allocated) using a computerised random number generator. A unique randomisation number will be linked with each treatment allocation. We will use blocking to ensure the required allocation ratio is maintained throughout the trial.

#### Concealment mechanism {16b}

The IT expert will send the randomisation list to the clinical trial supplies company so that blinded treatment packs can be prepared. The company will produce, label and package tranexamic acid and placebo ampoules into patient treatment packs as per the randomisation list. Tranexamic acid and placebo ampoules will look identical. The trial staff (coordinating centres and sites) will not have access to the randomisation list until after the final database lock.

#### Implementation {16c}

Once a woman is confirmed to be eligible, just prior to skin incision in caesarean section births (after draping), or at crowning in vaginal births, a treatment pack will be taken from a box of packs by a delegated member of the research team. The woman is considered to have been randomised once administration of the first IM injection has started. Each site will keep a log of women they enrol in the trial.

### Assignment of interventions: blinding

#### Who will be blinded {17a}

Trial participants, trial staff, healthcare providers, outcome assessors and data analysts will be blinded. The trial treatment packs will look identical except for the unique randomisation number. All women will receive two IM injections and one IV injection to ensure blinding of the route of tranexamic acid administration. The IM and IV treatment kits within each treatment pack will be clearly labelled to show the intended route of administration.

#### Procedure for unblinding if needed {17b}

There should be no need to unblind the allocated treatment. If a woman develops PPH, she should receive all clinically indicated treatments, which can include tranexamic acid. Because a second 1-g dose of tranexamic acid is well within the usual dosing range, it is not necessary to find out whether a particular woman received tranexamic acid or placebo as part of the I’M WOMAN trial. Even if a particular woman has received tranexamic acid within the trial, a second 1-g dose of tranexamic acid can safely be given. Nevertheless, if the clinician believes that a woman’s care depends importantly upon knowledge of whether the participant received tranexamic acid or placebo, it is possible to unblind. If urgent unblinding is necessary, the CTU will provide an emergency 24-h telephone service. The caller will receive a voice message, text message or email informing them whether the woman received tranexamic acid or placebo. The investigator should complete an unblinding request/report form within five working days of unblinding. If a suspected unexpected serious adverse reaction (SUSAR) is reported, unblinding may be needed for reporting to regulatory agencies and ethics committees.

### Data collection and management

#### Plans for assessment and collection of outcomes {18a}

The local trial staff will collect consent, baseline, outcome, and adverse event data and send them to the LSHTM CTU Global Health Trials Group by entering them into the online trial database. All staff authorised to collect trial data will be trained in data collection procedures and GCP. The staff will first enter trial data onto paper case report forms (CRF) and then the trial database to allow easier data control. After completing the consent procedure, an entry form will be used to collect baseline data including the woman’s age, gestational age, parity, recent Hb or PCV if known, multiple pregnancy, planned route of birth, previous PPH, hypertensive disease, placental abnormality, antepartum haemorrhage, and any other PPH risk factors. We will also collect the date and time of randomisation and IM injection sites. We will follow up participants until discharge, death, or the end of the postnatal period (42 days), whichever occurs first, and trained staff will record outcome data onto an outcome form.

#### Plans to promote participant retention and complete follow-up {18b}

Follow-up will take place in a hospital and should be minimal, so there is no need for strategies to increase retention. A woman can withdraw from the trial at any time. She may give her reason for withdrawal but she does not have to. If a woman withdraws from the trial, we will analyse data collected to the point of withdrawal, but no other data will be collected unless the woman gives permission. In all cases, we will respect the woman’s wishes. At discharge, women will be given an ‘alert card’ identifying them as an I’M WOMAN trial participant and asked to present this card to anyone providing medical care after discharge, up to day 42. The card will have instructions to ensure the AE reporting procedures are followed.

#### Data processing pathway {19}

Data will be collected at each site by local investigators and sent to the LSHTM CTU Global Health Trials Group. On completion of the paper CRF, data will be submitted by authorised investigators into the trial database held by the LSHTM CTU Global Health Trials Group, accessed via a unique username, password, and PIN. Data will have a one-pass data entry by investigators entering forms directly onto the trial database. One hundred per cent of data received will be subject to automatic electronic validation checks built into the database, such as checks of ranges and illogical values, and manual review, in line with the I’M WOMAN Database Development and Validation Plan and I’M WOMAN Data Management Plan. Missing data will be recorded and presented as such, not imputed. Correction of discordant data will be resolved through queries on the trial database via the inbuilt query management system, followed up regularly until resolved.

#### Coding

Coding will be completed manually, no auto-encoding will be carried out. The MedDRA coding dictionary will be used for medical terms reported as adverse events and cause of death, with the British National Formulary online used for concomitant medication data collected as part of adverse event reporting.

#### Data storage

Data stored on the trial database will be pseudonymised by linking to a unique participant ID number only. The name and any other identifying details will not be included in the trial data electronic file used for analysis or publication. A risk assessment for the trial database will be conducted, covering all the risks associated to the front-end and back-end security of the systems, outlined in the I’M WOMAN Database Development and Validation Plan. The LSHTM CTU Global Health Trials Group will provide an Investigator Site File (ISF) containing the essential trial documents, which the site must keep updated throughout the trial. Original copies of CRFs, consent forms, and source data will be kept securely at each participating site. These must be archived securely for 10 years after the overall end of the trial. Source documents include, but are not limited to, hospital records (from which medical history, previous and concurrent medication, clinical outcomes, and adverse events may be summarised onto CRFs), clinical and office logbooks, laboratory and pharmacy records, diaries, and correspondence. On all trial-specific documents other than the consent form, we will refer to the participant by their screening ID number and randomisation number. Study sites will provide data access to authorised representatives of the sponsor, the host institution, and regulatory authorities to allow trial-related monitoring, audits, and inspections.

#### Confidentiality {27}

The trial staff will ensure that participants’ confidentiality is maintained. Any identifiable data obtained by the LSHTM CTU Global Health Trials Group will be stored securely and confidentiality protected in accordance with relevant Data Protection regulations including the UK General Data Protection Regulation (GDPR) 2018. Participants will be identified only by a participant screening ID and randomisation number on all trial documents and any electronic database, except for the paper CRF which remains at participating sites, where participant initials may be added. All documents will be stored securely and only accessible by the trial staff and authorised personnel. The LSHTM CTU Global Health Trials Group will securely store copies of consent forms sent for monitoring, and these will be destroyed at trial closure. Only people authorised by the chief investigator, project lead, or trial manager will have access to the I’M WOMAN trial database. The trial database will be accessed through a complex password system which includes a password ageing mechanism (i.e. passwords will be changed every 90 days).

## Statistical methods

### Statistical methods for primary and secondary outcomes {20a}

We will draft a statistical analysis plan (SAP) for use by the Data Monitoring Committee (DMC) during their ongoing review. The SAP will be finalised before the trial database is locked for the final analysis.

The primary analysis comparing IM and IV tranexamic acid will be a per-protocol analysis. The analysis comparing tranexamic acid to placebo will be an intention-to-treat analysis. We will analyse the data and present statistics by randomised group. We will tabulate demographic and other baseline characteristics. Descriptive statistics for continuous variables will include the mean, standard deviation, median, range, and the number of observations. We will present categorical variables as numbers and percentages. Effect measures will be relative risk. Precision will be quantified using 95% confidence intervals. We will use the composite strategy to account for intercurrent events that may preclude the measurement of key efficacy and safety outcomes (e.g. early death, surgical interventions, discharge, or transfer to another hospital).

To compare the IV and IM routes of administration, we need to prespecify a non-inferiority margin—a predetermined margin of difference that is clinically acceptable. Women often experience treatment delays while waiting for a doctor or travelling to the hospital. Because tranexamic acid’s effectiveness falls by about 10% for every 15-min delay, a 1-h treatment delay corresponds to a 40% reduction in effectiveness. If IM administration preserves 60% of the benefit achieved with IV administration, IM tranexamic acid would be equivalent to an IV injection with a 1-h delay. An effect of this size from a more accessible route of administration with potentially fewer side effects would be clinically relevant. A 60% preservation fraction corresponds to a relative risk between IM and IV tranexamic acid of 1.13. If the upper limit of the two-sided 95% confidence interval for IM versus IV tranexamic acid is less than 1.13, then evidence of non-inferiority will have been provided.

### Interim analyses {21b}

The DMC is responsible for deciding whether to reveal the unblinded results (overall or for a particular subgroup) to the Trial Steering Committee (TSC) while the trial is underway. The DMC will do this only if two conditions are satisfied: (1) the results provide proof beyond reasonable doubt that treatment is either definitely harmful/inferior or definitely favourable for all or for a particular subgroup in terms of a major outcome and (2) the results are expected to substantially change the prescribing patterns of clinicians who are familiar with other existing trial results. The exact criteria for ‘proof beyond reasonable doubt’ are not and cannot be specified by a purely mathematical stopping rule but are strongly influenced by such rules. The DMC Charter will refer to the Peto-Haybittle stopping rule, whereby an interim analysis of a major outcome must involve a difference between the treatment and control of at least three standard errors to justify premature disclosure [33, 34]. An interim subgroup analysis would have to be even more extreme to justify disclosure.

We will present baseline data, outcome data, and data on adverse events that might be associated with postpartum bleeding or tranexamic acid use to the DMC. We will use the Peto-Haybittle stopping rules, which require extreme differences to justify premature disclosure and involve an appropriate combination of mathematical stopping rules and scientific judgement, with the advantage that the exact number and timing of interim analyses need not be pre-specified. If there is a difference of at least three standard errors in favour of tranexamic acid compared to placebo, this will be taken as proof of a treatment benefit, and we will stop recruitment into the placebo group. The trial will continue to assess the non-inferiority of IM tranexamic acid compared to IV tranexamic acid. While early stopping based on evidence of non-inferiority is not recommended, early stopping based on inferiority is recommended to minimise patient exposure to an inferior intervention. The Peto-Haybittle stopping rule is appropriate as both the treatment and active control are the same drug given via different routes, with no good biological reason to expect the inferiority of the IM route. If there is a difference of at least three standard errors in favour of IV tranexamic acid compared to IM tranexamic acid, this will be taken as proof of inferiority, and we will stop recruitment into the IM tranexamic acid group.

### Methods for additional analyses (e.g. subgroup analyses) {20b}

In a large trial such as I’M WOMAN, baseline characteristics of participants that may influence the outcome should be evenly distributed between the treatment and placebo groups, so that any difference in outcome can be attributed to the intervention. However, it is still possible that a chance imbalance in important prognostic factors could influence the results. To investigate this possibility, an analysis adjusted for baseline risk will be conducted. A prognostic model will be built based on pre-specified baseline variables and used to estimate the predicted risk of the outcome at baseline.

Planned subgroup analyses will be conducted on the primary outcome comparing tranexamic acid to placebo. Subgroup variables include severity of anaemia (no anaemia vs mild anaemia vs moderate/severe anaemia) and route of birth (vaginal vs caesarean). We will explore potential confounding in subgroup analyses. Randomisation creates treatment groups that are balanced; however, the strata of subgroups may not be balanced [35]. It is possible that some baseline variables will be associated with the subgroup variable and the treatment effect. We will investigate the association of anaemia and route of birth with other baseline variables and adjust for any potential confounders as necessary. We will report relative risks (RR) and confidence intervals alongside *p*-values from tests for interaction. Unless there is strong evidence of interaction (*p* < 0.001), we will take the overall RR as the most reliable estimate of the RR in all subgroups.

### Methods in analysis to handle protocol non-adherence and any statistical methods to handle missing data {20c}

We will conduct both per-protocol and intention-to-treat analyses. We will conduct a sensitivity analysis excluding participants in the IM and IV tranexamic acid groups who did not receive tranexamic acid. Because we expect missing data to be minimal, we will summarise missing data for baseline and outcome variables but will not use any methods to impute it.

### Plans to give access to the full protocol, participant-level data, and statistical code {31c}

The anonymised, participant-level trial dataset and statistical code will be shared via the LSHTM CTU Global Health Trials Group data sharing platform at freebird.lshtm.ac.uk. All trial materials including training materials, CRFs, and full protocol will be made available on the trial website and team YouTube channel.

## Oversight and monitoring

### Composition of the coordinating centre and trial steering committee {5d}

The trial involves screening, seeking consent, giving the trial treatment, and collecting baseline and outcome data (mostly from the hospital notes). A Trial Management Group (TMG) will oversee the trial progress, while the LSHTM CTU Global Health Trials Group will coordinate day-to-day trial management. The TMG will consist of the Protocol Committee members and the data manager. The TMG and trial manager will act on behalf of the sponsor and ensure that the sponsor’s responsibilities are carried out. These responsibilities include (but are not limited to) the following: report to the TSC, maintain the Trial Master File, identify sites, assess site suitability, confirm all approvals are in place before enrolment of participants and release of the trial treatment, provide training, provide study materials, data management, 24-h unblinding service, monitoring, ensure data security and quality and observe data protection laws, safety reporting, ensure trial is conducted in accordance with the ICH GCP, progress updates, respond to questions about the trial, statistical analysis, and publication of trial results.

The Protocol Committee will be responsible for developing the protocol. Subsequent changes to the final protocol will require the agreement of the TSC. Members will be included as authors on the final published protocol.

The TSC will include (but is not limited to) an independent chair, experienced obstetrician, clinical trialist, clinical representative from a low- and middle-income country (LMIC), a statistician, lay representative, and some members of the TMG (see Additional file 7). The TSC will supervise the trial and advise the sponsor, with a focus on trial progress, protocol adherence, participant safety, and consideration of new information. The TSC must agree on the final protocol and, throughout the trial, take responsibility for major decisions such as protocol amendments, monitoring and supervising trial progress, reviewing relevant information from other sources, considering recommendations from the DMC, informing and advising the TMG. In general, we will aim to hold meetings about once per year unless there is a need to hold them more often. A TSC Charter agreed upon at the first meeting will detail the conduct of business.

We will identify a National Coordinating Investigator for each participating country, who will be responsible for ensuring that all national approvals including those from regulatory agencies, ethics committees and relevant import licences are in place before the trial starts in their country. Additionally, they will support the LSHTM CTU Global Health Trials Group by ensuring recruitment is on target, safety reporting to all relevant agencies, and site training and monitoring as required.

A site principal investigator will coordinate the trial at each participating hospital. Site-specific responsibilities detailed in an agreement in advance of starting the trial will include the following:

- Supervise the study at their site, comply with the final trial protocol and amendments, obtain all appropriate approvals, and account for trial treatments
- Ensure the trial is conducted in line with ICH GCP and fulfils all national and local regulatory requirements
- Keep the trial staff aware of the current state of knowledge, the trial, and its procedures (there are training materials to assist with this)
- Delegate trial responsibilities to suitably trained, qualified personnel and document delegation
- Ensure that all potentially eligible women are considered promptly for the trial and consent is obtained in line with local approved procedures
- Ensure that the data are collected, completed, and sent to the CTU Global Health Trials Group in a timely manner, including adverse events reporting
- Ensure the Investigator Study File is up-to-date and complete
- Allow access to source data, including participants’ medical records, for monitoring, audit, and inspection
- Be responsible for archiving all original trial documents including medical records, Investigator Study File, consent forms, and data forms for at least 10 years after the end of the trial

The trial will be conducted in accordance with the current approved protocol, GCP, relevant regulations guidance provided in the ISF, and the trial’s standard operating procedures. A detailed monitoring plan will be developed. In summary, the LSHTM CTU Global Health Trials Group will closely monitor the trial to ensure the rights, safety, and well-being of the trial participants and the accuracy of the data. All coordinating centres and site teams will be trained in the trial procedures and provided with extensive guidance. We will use central monitoring methods. A sample of consent forms from all sites will be monitored to check they are properly completed. Data management and statistical checks (central statistical monitoring) will ensure the inclusion criteria are met and the trial treatment is administered in line with the protocol. Outcome event rates will be monitored. Quantitative variables will be monitored to check data validity using statistical methods such as the coefficient of variation and runs test. Sites with unusual event rates or low variability or randomness in the data will be selected for further monitoring.

Sites flagged as high risk by central monitoring procedures may require onsite monitoring with source data verification. Site self-monitoring will also be carried out where needed. The site PI/delegate will monitor themselves against a standardised checklist. Site investigators and their institutions will provide access to source data and all trial-related documents for monitoring, audits, ethics committee review, and regulatory inspection. All trial-related and source documents including medical records, original consent forms, and original CRFs must be kept safely. Investigators must plan in advance of the trial start where the trial-related documents will be stored and how they will be accessed. All documents must be made available for up for 10 years after the end of the overall trial.

Deviations from clinical trial protocols and GCP occur commonly in clinical trials. A protocol deviation is a departure from the approved protocol’s procedures made with or without prior approval. Such departures may be major or minor/administrative. Most deviations do not result in harm to trial subjects or affect the scientific value of the trial. All deviations must be reported to the LSHTM CTU Global Health Trials Group within 24 h of it becoming known to the trial team.

A serious breach is defined as ‘a breach of GCP or the trial protocol which is likely to affect to a significant degree (a) the safety or physical or mental integrity of the subjects of the trial or (b) the scientific value of the trial’. If suspected, the site should inform the LSHTM CTU Global Health Trials Group within 24 h. The LSHTM CTU Global Health Trials Group will report all serious breaches to the relevant regulatory authorities and REC within the timeline required by each participating country.

### Composition of the data monitoring committee, its role, and reporting structure {21a}

The sponsor is primarily responsible for monitoring the safety of participants in the trial, overseen by an independent DMC to support the safety monitoring. Membership includes expertise in maternal health, statistics, and study design (see Additional file 6). The DMC is independent from the sponsor. The DMC will review accumulating trial data and advise the TSC on the continuing safety of trial participants and those yet to be recruited. The DMC Charter will list the composition, name, title, and address of the chairperson and DMC members, in line with the DAMOCLES Study Group recommendations [36]. The DMC Charter will also include the schedule and format of the DMC meetings, format for data presentation, reporting method, timing of interim reports, and stopping rules. The DMC is independent of the sponsor, ethics committees, regulatory agencies, investigators, steering committee membership, clinical care of the trial participants, and any other capacity related to trial operations.

### Adverse event reporting and harms {22}

Maternal and neonatal events which occur as a consequence of the CS or vaginal birth, events which commonly occur in this population independent of the trial treatment, events which are present before randomisation, and events which are recorded as study outcomes, do not need to be reported as adverse events. Although congenital and genetic abnormalities cannot be attributed to the trial treatment because it is given minutes before birth, we will record these on the outcome form.

Events recorded on the outcome form up to discharge, death, or day 42 (whichever is sooner) will be presented to the DMC for regular review, and so will not be reported using the adverse events (AE) reporting procedure. For maternal outcomes, this includes PPH, nausea, vomiting, dizziness, vascular occlusive events, seizure, infection, sepsis, organ dysfunction, and pain or adverse skin reaction at the injection site. For neonatal outcomes, this includes stillbirth/intrapartum death, neonatal death, intracranial and pulmonary haemorrhage, bruising, seizure, and vascular occlusive events.

Other medical events that fulfil the AE definition below will be reported up to 42 days after administration of trial treatment. If a woman is discharged before 42 days, outcome events after discharge and up to 42 days that fulfil the AE definition will be reported, including those on the outcome form. At discharge, women will be given an ‘alert card’ identifying them as an I’M WOMAN trial participant and asked to present this card to anyone providing medical care after discharge, up to day 42. The card will have instructions to ensure the AE reporting procedures are followed. A safety reporting overview is provided in Additional file 5.

#### Definitions

Adverse event (AE): Any untoward medical occurrence in a participant to whom a medicinal product has been administered including occurrences which are not necessarily caused by or related to that product. An AE can, therefore, be any unfavourable and unintended sign (including an abnormal laboratory finding), symptom, or disease temporally associated with the use of an IMP.

Adverse reaction (AR): Any untoward and unintended response in a participant to an IMP which is related to any dose administered to that participant. The phrase ‘response to an investigational medicinal product’ means that a causal relationship between a trial medication and an AE is at least a reasonable possibility, i.e. the relationship cannot be ruled out.

Serious adverse event (SAE): A SAE is any untoward medical occurrence that results in death, is life-threatening, requires inpatient hospitalisation or prolongation of existing hospitalisation, and results in persistent or significant disability/incapacity; other ‘important medical events’ may also be considered serious if they jeopardise the participant or require an intervention to prevent one of the above consequences.

Serious adverse reaction (SAR): An AE that is both serious and, in the opinion of the reporting investigator, believed with reasonable probability to be due to the trial treatments, based on the information provided.

Suspected unexpected serious adverse reaction (SUSAR): A serious adverse reaction, the nature and severity of which is not consistent with the information about the medicinal product in question set out in the in the Investigator’s Brochure (IB).

When completing the adverse event reporting form, the site PI or medical delegate will assign a causality using the definitions below:

- Suspected to be related—There is evidence to suggest a causal relationship with the administration of the trial treatment and the influence of other factors is unlikely.
- Not suspected to be—There is little or no evidence to suggest there is a causal relationship (e.g. the event did not occur within a reasonable time after administration of the trial treatment). There is another reasonable explanation for the event (e.g. the participant’s clinical condition, other concomitant treatment).

If there is any doubt about the causality, the site PI or medical delegate will inform the LSHTM CTU Global Health Trials Group. In the case of discrepant views on causality between the investigator and others, all parties will discuss the case. If no agreement is made, both points of view will be recorded and reported onwards as required.

#### Reporting procedures

##### Adverse reactions (ARs)/adverse events (AEs)

Site investigators will report non-serious ARs and AEs using the AE reporting forms provided to them.

##### Serious adverse reactions (SARs)/serious adverse events (SAEs)

The site principal investigator (PI) or medical delegate must report AEs and ARs that fulfil the serious criteria to the LSHTM CTU Global Health Trials Group within 24 h of becoming aware of the event using the AE reporting form. The site PI or medical delegate will complete the form with as much detail as is available. A follow-up report will be submitted promptly should any additional information arise (but no later than five working days of becoming aware of the event). The site PI or medical delegate will record an assessment of seriousness, causality, and expectedness. Events relating to a pre-existing condition or any planned hospitalisations for elective treatment of a pre-existing condition will not be reported as SAEs.

##### Suspected unexpected serious adverse reactions (SUSARs)

All SAEs assigned by the site PI or medical delegate as suspected to be related to the trial treatment and which are unexpected will be classified as suspected, unexpected, serious adverse reactions (SUSAR) and will be subject to expedited reporting to each participating Regulatory Authority, Ethics Committees and the sponsor within seven working days of being reported to the LSHTM CTU Global Health Trials Group.

In the case of a SUSAR, the site staff will:

1. Contact the LSHTM CTU Global Health Trials Group immediately by phone or email to inform them of the event and obtain guidance on the reporting procedure if needed
2. Submit an AE report, completed with all available information (signed and dated) within 24 h, together with relevant treatment forms and anonymised copies of all relevant clinical investigations
3. Submit any additional information promptly upon request

Emergency contact details for advice on reporting SAEs and SUSARs can be found in the Investigator Study File. AE reporting forms will be submitted either via the trial database (see Investigator Study File for full details) or email to imwoman.data@lshtm.ac.uk. AEs that the site PI or the LSHTM CTU Global Health Trials Group consider related to the trial medication will be followed either until resolution or the event is considered stable.

The LSHTM CTU Global Health Trials Group or sponsor representative will report all SUSARs to the relevant regulatory authorities, Research Ethics Committees (REC), and other parties as applicable. For fatal and life-threatening SUSARS, this will be done no later than seven calendar days after the LSHTM CTU Global Health Trials Group is first made aware of the reaction. Any additional relevant information will be reported within eight calendar days of the initial report. All other SUSARs will be reported within 15 calendar days. Treatment codes will be unblinded for specific participants if required. Site PIs will be informed of all SUSARs for all studies sponsored by LSHTM that use tranexamic acid, whether the event occurred in the I’M WOMAN trial. All other AEs will be reported as requested by the relevant authorities.

### Frequency and plans for auditing trial conduct {23}

The study may be subject to audit by LSHTM under their legal obligation as sponsor. Additionally, inspections can be carried out by relevant REC and regulatory authorities to ensure adherence to the protocol, Good Clinical Practice, relevant regulations, and funder requirements.

### Plans for communicating important protocol amendments to relevant parties (e.g. trial participants, ethical committees) {25}

All changes to the protocol will require the agreement of the Trial Steering Committee (TSC). We will notify the sponsor of agreed amendments to decide if the amendment is substantial or not. The chief investigator or delegate will ensure all amendment notifications and associated documents are updated and submitted to the relevant parties (e.g. sites, investigators, REC, institutional review boards (IRB), trial participants, trial registries, journals, regulators). All participating sites affected will be notified of the amendment in writing. All documentation relating to the amendment will be filed in the Trial Master File (TMF) and ISF.

## Dissemination plans {31a}

Publications will only contain anonymised data. We aim to publish the main results of the I’M WOMAN trial in a peer-reviewed journal under a CC-BY License. This license will ensure the publication is freely available and can be distributed by others if they give credit to the original creation. The main publication will follow the Consolidated Standards of Reporting Trials (CONSORT) statement. Links to publications will be made in any applicable trial registers. The results will be disseminated via social media, press, trial website, WOMAN trials newsletter, briefing papers, and relevant maternal health organisations.

The success of the trial will be dependent entirely upon the collaboration of healthcare professionals in the participating sites. Hence, the chief credit for the study will be assigned to the collaborators from each participating centre and they will be named personally in the main publications. The results of the trial will be reported first to trial collaborators. The main publication of the trial results will be in the name of the Trial Collaborative Group (I’M WOMAN trial collaborators).

## Discussion

The results of the I’M WOMAN trial will provide evidence of the benefits and harms of TXA for PPH prevention and the effects of the IM and IV routes of administration. The IM route should be as effective as the IV route for preventing bleeding. There may be fewer side effects with IM TXA because peak blood concentrations are lower than with the IV route. IM TXA also has practical advantages as it is quicker and simpler to administer. By avoiding the need for IV line insertion and a slow IV injection, IM administration would free up overstretched midwives and doctors to focus on looking after the mother and baby and expand access to timely TXA treatment.

## Trial status

The current protocol is version 2.0, dated 1 June 2023. Recruitment is planned to start by December 2023. The end of recruitment is planned for November 2025. Further information is available at https://imwoman.lshtm.ac.uk/. For the latest news and updates, you can subscribe to the WOMAN Trials newsletter here.

### Supplementary Information

**Additional file 1.** Main Contacts.

**Additional file 2.** Consent Overview.

**Additional file 3.** Brief Information Sheet.

**Additional file 4.** Participant Information Sheet and Informed Consent Form.

**Additional file 5.** Safety Reporting Overview.

**Additional file 6.** Data Monitoring Committee Membership.

**Additional file 7.** Trial Steering Committee Membership.

**Additional file 8.** List of Major Risk Factors for Postpartum Haemorrhage.

## Data Availability

We are committed to sharing data for ethical research with justified scientific objectives. Until all planned analyses are completed by the LSHTM CTU Global Health Trials Group, data will be shared through a controlled access approach whereby researchers can make formal applications for data sharing. Afterwards, the anonymised dataset will be shared via the LSHTM CTU Global Health Trials Group data sharing platform at freebird.lshtm.ac.uk. All trial materials including training materials, CRFs, and protocol will be made available on the trial website and team YouTube channel.

## Abbreviations

AE: Adverse event
AR: Adverse reaction
CONSORT: Consolidated Standards of Reporting Trials
CRF: Case report form
CS: Caesarean section
CTU: Clinical Trial Unit
DAL: Drug Accountability Log
DDF: Drug destruction form
DMC: Data Monitoring Committee
GCP: Good Clinical Practice
GDPR: General Data Protection Regulation
GMP: Good Manufacturing Practice
Hb: Haemoglobin
HDU: High dependency unit
IB: Investigator’s Brochure
ICH: International Council for Harmonisation of Technical Requirements for Pharmaceuticals for Human Use
ICH GCP: ICH Good Clinical Practice
ICU: Intensive care unit
EMA: European Medicines Agency
IM: Intramuscular
IMP: Investigational medicinal product
IRB: Institutional Review Board
ISF: Investigator Site File
IV: Intravenous
LMICs: Low- and middle-income countries
LSHTM: London School of Hygiene and Tropical Medicine
PCV: Packed cell volume
PI: Principal investigator
PPH: Postpartum haemorrhage
QP: Qualified person
REC: Research Ethics Committee
RR: Relative risk
SAE: Serious adverse event
SAP: Statistical analysis plan
SAR: Serious adverse reaction
SUSAR: Suspected unexpected serious adverse reaction
TMF: Trial Master File
TMG: Trial Management Group
TSC: Trial Steering Committee
TXA: Tranexamic acid
WHO: World Health Organization
