# Supplementary material for: Tranexamic acid by the intramuscular or intravenous route for the prevention of postpartum haemorrhage in women at increased risk: a randomised placebo-controlled trial (I’M WOMAN)
Source: Trials. 2023 Dec 3;24:782. doi: 10.1186/s13063-023-07687-1 (PMC10694937; doi:10.1186/s13063-023-07687-1)
Supplement: Supplementary file 1 — Additional file 1. Main Contacts. [file 13063_2023_7687_MOESM1_ESM.pdf]

Appendix 1 – Main Contacts

**LSHTM Clinical Trials Unit – Global Health Trials Group**	I'M WOMAN Trial<br>Clinical Trials Unit – Global Health Trials Group<br>London School of Hygiene & Tropical Medicine,<br>Keppel Street<br>London, WC1E 7HT, UK<br>Tel: +44(0)20 7299 4684<br>Email: IMWOMAN@lshtm.ac.uk<br>Web: www.imwoman.lshtm.ac.uk
**Pakistan Clinical Trials Unit**	GIHDSTMU-LSHTM Research Collaboration Centre<br>Global Institute of Human Development<br>Shifa Tameer-e-Millat University, STMU Campus<br>Shifa International Hospital<br>Pitras Bukhari Road, Sector H-8/4<br>Islamabad, Pakistan<br>Tel: +44 (0) 2072994837<br>Email: pakistan.imwoman@lshtm-ctu.org
**Nigeria Clinical Trials Unit**	COMUI-LSHTM Research Collaboration Centre<br>College of Medicine<br>University of Ibadan<br>Queen Elizabeth Road<br>Ibadan, Nigeria<br>Tel: +44(0)20 7958 8517<br>Email: nigeria.imwoman@lshtm-ctu.org
**Tanzania Clinical Trials Unit**	UDSM-LSHTM Research Collaboration Centre<br>University of Dar es salaam, Mbeya College of Health and allied Sciences<br>P.O.Box 35091<br>Dar es Salaam, Tanzania<br>Tel: +255252500082<br>Email: tanzania.imwoman@lshtm-ctu.org
**Sponsor**	Research Governance & Integrity Office<br>London School of Hygiene & Tropical Medicine,<br>Keppel Street<br>London, WC1E 7HT, UK<br>Phone: +44 (0)20 7927 2626<br>Email: RGIO@lshtm.ac.uk
**Emergency telephone**	This emergency number is to be used only in the event urgent unblinding of the trial treatment:<br><br>+44(0)7768 707500
