# Supplementary figures and images for: Tranexamic acid by the intramuscular or intravenous route for the prevention of postpartum haemorrhage in women at increased risk: a randomised placebo-controlled trial (I’M WOMAN)
Source: Trials. 2023 Dec 3;24:782. doi: 10.1186/s13063-023-07687-1 (PMC10694937; doi:10.1186/s13063-023-07687-1)

## APPENDIX 2 - CONSENT PROCEDURE OVERVIEW

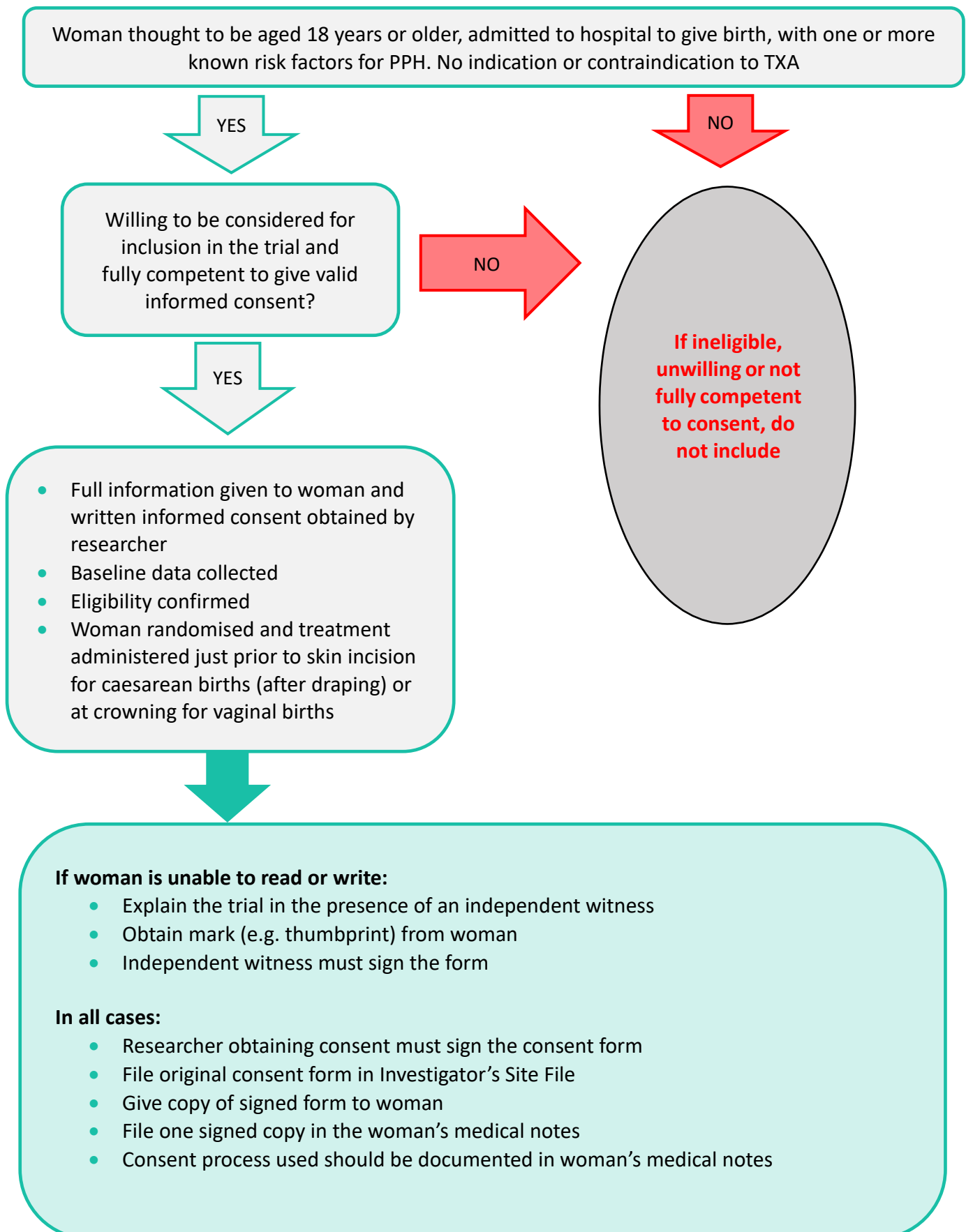

Supplement: Supplementary file 2 — Additional file 2. Consent Overview. [file 13063_2023_7687_MOESM2_ESM.pdf]

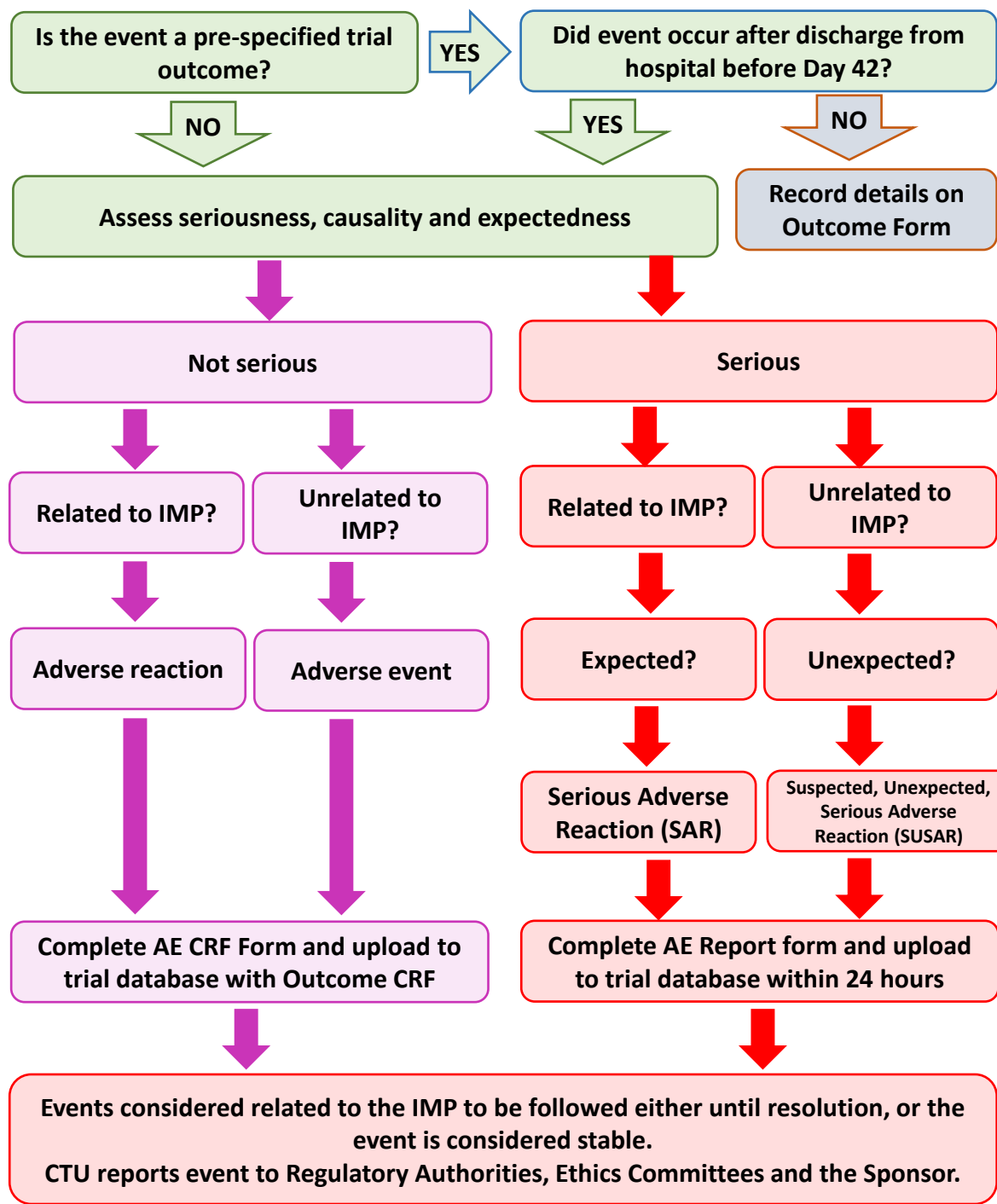

Supplement: Supplementary file 5 — Additional file 5. Safety Reporting Overview. [file 13063_2023_7687_MOESM5_ESM.pdf]
