# Supplementary material for: Tranexamic acid by the intramuscular or intravenous route for the prevention of postpartum haemorrhage in women at increased risk: a randomised placebo-controlled trial (I’M WOMAN)
Source: Trials. 2023 Dec 3;24:782. doi: 10.1186/s13063-023-07687-1 (PMC10694937; doi:10.1186/s13063-023-07687-1)
Supplement: Supplementary file 3 — Additional file 3. Brief Information Sheet. [file 13063_2023_7687_MOESM3_ESM.pdf]

**BRIEF STUDY INFORMATION SHEET**
**THE I'M WOMAN TRIAL**

(NOTE: final version will contain locally relevant, culturally sensitive images. These will be approved by the local REC before use).

This hospital is taking part in a research study to find ways to prevent heavy bleeding after childbirth. This leaflet explains why we are doing this study and what it involves.

**What is the I'M WOMAN trial?**
The I'M WOMAN trial is a study to see if a drug called tranexamic acid (TXA) can prevent heavy bleeding after childbirth when it is given into the muscle. About 30,000 women giving birth in hospitals around the world will be taking part. It has been approved by ethics and regulatory agencies in your country.

**What is heavy bleeding after childbirth?**
Vaginal bleeding after childbirth is normal. It usually stops on its own and is nothing to worry about. But some women have heavy bleeding – this is called a postpartum haemorrhage or PPH. A PPH can make women very unwell and is sometimes life-threatening.

**What is tranexamic acid?**
TXA is a drug that reduces bleeding. It is not a new drug. It is often used to reduce bleeding in operations and after serious injury. In an earlier study, we gave TXA to thousands of women who were having a PPH. It saved the lives of about 1 in 3 women who had a PPH, and it did not cause any serious side effects. The WHO recommends that all women who are having a PPH get TXA.

**Why are we doing this study?**

Our previous studies show that TXA is most effective when given early. This made us wonder if giving TXA before the birth of the baby might prevent PPH from happening in the first place. Preventing PPH might be better than treating a PPH after it happens. This study will find out if TXA can prevent PPH from happening.

TXA is usually given into a vein. But it can also be given into a muscle, like a vaccine. Giving TXA into a muscle is easier and quicker. We hope this study proves that both ways of giving TXA are equally good at preventing PPH. TXA sometimes causes mild side effects like feeling sick. This may be less likely if TXA is given into the muscle. We also hope the study proves this.

**What does the study involve?**
Taking part will not affect how you plan to have your baby. You will get all the usual care for women giving birth at your hospital. The study treatment is free. It will not cost you any money to take part.

We will review your medical records and may ask you some questions to see if you are at higher risk of PPH. If you are, you will be invited to take part. If you agree, just before your baby is born, you will receive two injections into different muscles and one into a vein. The injections will hold either TXA or placebo (a dummy drug that is completely safe). What each injection holds is decided randomly. After you give birth, we will measure how much blood you lose and collect information on how you and your baby are getting on.

A small amount of the medicine might cross over to the baby through the placenta or breast milk. Earlier studies did not find any harmful effects in babies whose mothers who got TXA when they were pregnant, or who were breastfed by mothers who got TXA. TXA has been used in adults, children, and babies for many years without any harmful effects.

We will give you a card with contact details of the study doctor at this hospital, which you can show to doctors if you return to hospital, so that they know were in the study. If you return to hospital for any medical problem related to the study, we will pay your travel costs.

If you want more information about the study, the study coordinators at this hospital can be contacted on:
Name
Address
Phone
Email

The study is organised by the London School of Hygiene & Tropical Medicine (University of London) and is supervised in [Country] by xxxx.  You can also contact them directly for information about the trial.
Name of NCC
Address
Phone
Email
