# Supplementary material for: Tranexamic acid by the intramuscular or intravenous route for the prevention of postpartum haemorrhage in women at increased risk: a randomised placebo-controlled trial (I’M WOMAN)
Source: Trials. 2023 Dec 3;24:782. doi: 10.1186/s13063-023-07687-1 (PMC10694937; doi:10.1186/s13063-023-07687-1)
Supplement: Supplementary file 4 — Additional file 4. Participant Information Sheet and Informed Consent Form. [file 13063_2023_7687_MOESM4_ESM.zip › Appendix 4a PIS IMW v1.2 15May23_CleanR0.pdf]

[Hospital Contact Details]
Name of PI
Name of Hospital
Hospital address
Contact phone number

The I'M WOMAN trial

Intramuscular tranexamic acid to prevent heavy bleeding after childbirth

in women at higher risk

(NOTE: final version will contain locally relevant, culturally sensitive images. These will be approved by the local REC before use).

STUDY INFORMATION FOR PARTICIPANTS

We invite you to take part in a research study called I'M WOMAN

- Before you decide, we want you to know why the study is being done and what it involves.
- Please read this information or ask the doctors or midwifes to explain it to you. Ask as many questions as you like before deciding whether to take part.
- Taking part is your decision. If you choose not to take part, the doctors and midwives will give you all the usual care given at this hospital.

Contents

1. What is the study for?
2. Why are you asking me to take part?
3. What will happen if I take part?
4. How long will I be in the study?
5. Will I benefit from taking part?
6. Could I be harmed by taking part?
7. Can I change my mind about taking part?
8. What happens afterwards?
9. What information do we keep private?
10. Who is doing this study?
11. Who has reviewed the study?
12. Who can I contact about any questions?
13. What if there is a problem?
14. What else do I need to know?

1. What is the study for?

Vaginal bleeding after childbirth is normal. It usually stops on its own and is nothing to worry about. But some women have heavy bleeding - this is called a postpartum haemorrhage or PPH. A PPH can make women very unwell and is sometimes life-threatening. In this study, we are looking to see if a drug called tranexamic acid (TXA) can prevent PPH when it is given into the muscle.

TXA is a drug that reduces bleeding. It is not a new drug. It is often used to reduce bleeding in operations and after serious injury. In an earlier study, we gave TXA to thousands of women who were having a PPH. It saved the lives of about 1 in 3 women who had a PPH, and it did not cause any serious side effects. The WHO (World Health Organisation) recommends that all women who are having a PPH get TXA.

Our previous studies show that TXA is most effective when given early. This made us wonder if giving TXA before the birth of the baby might prevent PPH from happening in the first place. Preventing PPH might be better than treating a PPH after it happens. This study will find out if TXA can prevent PPH from happening.

TXA is usually given into a vein. But it can also be given into a muscle, like a vaccine. Giving TXA into a muscle is easier and quicker. We hope this study proves that both ways of giving TXA are equally good at preventing PPH. TXA sometimes causes mild side effects like feeling sick. This may be less likely if TXA is given into the muscle. We also hope the study proves this.

2. Why are you asking me to take part?

We are asking you to take part because your doctor thinks that you have a higher chance of PPH. You need to be 18 years or older to take part. About 30,000 women around the world will be taking part in this study. Whether you take part is your decision.

3. What will happen if I take part?

Taking part will not change how you plan to have your baby. You will get all the usual care for women giving birth at your hospital. The study treatment is free. It will not cost you any money to take part.

If you want to take part, we will ask you to fill in a consent form. Then we will collect information about you and your labour. Just before your baby is born, you will be given two injections into different muscles and one into a vein, which will contain either TXA or placebo (a dummy drug that is completely safe). What each injection holds is decided randomly. The study drug and the placebo look the same, so you and your doctors will not know which you got. After you give birth, we will measure how much blood you lose and collect information about your health and your baby while you are in hospital.

4. How long will I be in this study?

You will be in the study until you leave hospital, or for six weeks after you had your baby, whichever is sooner. If you or your baby become ill after leaving hospital and within six weeks of giving birth, please tell the doctor named on this form.

5. What are the benefits of taking part?

We do not know if taking part in this study will help you personally or not. We hope that TXA will reduce the amount of blood women lose in childbirth and prevent PPH. If you take part, you may get TXA but there

is a chance you won't (i.e. if you are in the placebo group). If you don't take part, you won't get TXA before childbirth as it is not currently recommended or given in hospital to prevent PPH. By taking part, we hope you will be better informed for the future and can share your knowledge with others.

In the future, what we learn from this study will help doctors care for women who are having a PPH or who are at higher risk of having a PPH. If TXA prevents PPH and causes less side effects when it is given into the muscle, this will help women who give birth in situations where having an injection into a vein is not possible – an injection of TXA into the muscle could save their lives.

6. Could I be harmed by taking part?

TXA is widely used. The WHO recommends TXA for women who are having a PPH. Lots of studies with thousands of people suggest that TXA has clear health benefits and no serious side effects. Sometimes TXA can cause minor side effects like feeling or being sick (nausea), diarrhoea, and dizziness. Studies suggest that giving TXA into a vein or a muscle has no serious side effects. There is a small risk of redness, pain, or bruising at the injection sites. Injections have a very rare risk of infection.

A small amount of TXA can cross over to the baby through the placenta or breast milk. Earlier studies did not find any harmful effects in babies whose mothers who got TXA when they were pregnant, or who were breastfed by mothers who got TXA. Your doctor will watch you and your baby and give you the best available care if there are any problems. They will also tell the people running the study if there are any problems.

If TXA reduces the amount of blood you lose after childbirth, this will not harm your health. Bleeding after childbirth has no benefits, losing too much blood can make you ill, and PPH can be dangerous.

7. Can I change my mind about taking part?

Yes. You can stop taking part in the study at any time. You just need to say something like, *"I've decided I don't want to be in this study"*. Your doctor and the hospital staff will still care for you in the usual way. If you have any medical problems after you stop taking part, we ask that you still tell us about them.

8. What happens afterwards?

We will give you a card with the contact details of the study doctor at this hospital. Please keep this card safe. If you become ill within six weeks of having your baby, please contact the study doctor on the card. Please show this card to anyone who treats you for any illness within six weeks of having your baby.

If you would like to know the results of this study, please let the study doctor know and they will make sure you get a copy of the results. You can also visit the study website to keep up to date with progress: [insert website]

9. What information do we keep private?

We will keep all information collected about you and your baby private and stored securely. The only people allowed to look at the information are the staff running the study at the London Coordinating Centre and the [Name] Coordinating Centre in your country, as well as regulatory authorities who check that the study is being carried out correctly. The London Coordinating Centre may want to collect or copy some information with your name on it such as the consent form. These will be destroyed, or your personal details removed immediately after use.

We will make the study results public so doctors, midwives and researchers can learn from the study. We will not include your personal information in any study reports, so you will not be identified. The study team may share study data with other researchers and the public but will remove all personal information first.

10. Who is doing this study?

An international group of doctors, nurses, midwives and researchers are working together to find ways to improve women's health worldwide. The study is coordinated by a team of researchers at the London School of Hygiene & Tropical Medicine (University of London) in the United Kingdom and is supervised in [Country] by [Professor/Doctor name].

Name:	
Address:	
Phone:	
Email:	

11. Who has reviewed the study?

The WHO and an independent group of people called a Research Ethics Committee [Name] have carefully checked this study and agreed that it is okay for us to do it.

Name:			
Address:			
Telephone:		Fax:	
Website:			

12. Who can I contact about any questions?

If you have any questions about the study, ask to speak with the study team who will do their best to help. The contact details for the doctor in charge of the study at this hospital are:

Name:	
Address:	
Telephone:	
Email:	

13. What if there is a problem?

If something goes wrong and you are harmed during the study, the London School of Hygiene & Tropical Medicine (the sponsor) would be responsible for claims for any non-negligent harm. If you wish to make a complaint, you can do this through the hospital's complaints procedure. Please ask the study doctors or midwives for details.

If you return to hospital for any medical problem related to the study, we will pay your travel costs. If your medical problem is directly caused by the study drug, the sponsor will pay for necessary treatment.

The sponsor is not responsible for medical expenses due to pre-existing medical conditions, underlying diseases, ongoing treatments, or negligence or wilful misconduct by you, the study doctor, study site or third parties.

14. What else do I need to know?

The study is sponsored by the London School of Hygiene and Tropical Medicine (University of London, UK) and funded by Unitaid. Neither of these institutions make TXA.

Signing the consent form does not affect your legal rights to seek compensation.
