# Supplementary material for: Tranexamic acid by the intramuscular or intravenous route for the prevention of postpartum haemorrhage in women at increased risk: a randomised placebo-controlled trial (I’M WOMAN)
Source: Trials. 2023 Dec 3;24:782. doi: 10.1186/s13063-023-07687-1 (PMC10694937; doi:10.1186/s13063-023-07687-1)
Supplement: Supplementary file 4 — Additional file 4. Participant Information Sheet and Informed Consent Form. [file 13063_2023_7687_MOESM4_ESM.zip › Appendix 4b Consent form IMW v1.2 15May23_cleanR0.pdf]

[Hospital Contact Details]
Name of PI
Name of Hospital
Hospital address
Contact phone number
Email

# CONSENT FORM

## THE I'M WOMAN TRIAL

**Title of research: Intramuscular tranexamic acid to prevent heavy bleeding after childbirth in women at higher risk**

| | | | |
|---|---|---|---|
| Site ID Number | | Name of Site Principal Investigator | |
| Participant Hospital ID number | | Screening ID Number | |
| Name of Participant | | | |

**STATEMENT OF PERSON GIVING CONSENT:**

1. I confirm that I have read/have had read to me the information sheet for the above study in a language I understand.
2. I have discussed with the doctor to my satisfaction, and I have had the opportunity to ask questions.
3. I understand that my participation is voluntary. I have been given enough information about the research study to judge that I want to take part in it.
4. I understand that I am free to withdraw at any time, without giving any reason and without my medical care or legal rights being affected.
5. I understand that I will be given a copy of this consent form and the information sheet to keep for myself.
6. I understand that the study staff may look at sections of my medical notes and those of my baby/ies. I give permission for these individuals to have access to these records.
7. I understand that my data (with all personal information removed) will be made freely available for the public.
8. I give permission for a copy of this consent form, which contains my personal information, to be made available to the Trial Coordinating Centre in London for monitoring purposes only.
9. I agree to take part in the above study, the I'M WOMAN trial.

| | | |
|---|---|---|
| ______________________________ | ________________ | ______________________________________ |
| Name of woman | Date | Signature / Thumbprint or other mark (if unable to sign |
| ______________________________ | ________________ | ______________________________________ |
| Name of witness | Date | Signature |

*(A witness is needed if a patient cannot read or write)*

**STATEMENT OF PERSON OBTAINING INFORMED CONSENT:**

I have fully explained this research to this participant and have given sufficient information, including about risks and benefits, to make an informed decision.

| | | |
|---|---|---|
| ________________________ | ________________ | ______________________________ |
| Name | Date | Signature |
