# Supplementary material for: Tranexamic acid by the intramuscular or intravenous route for the prevention of postpartum haemorrhage in women at increased risk: a randomised placebo-controlled trial (I’M WOMAN)
Source: Trials. 2023 Dec 3;24:782. doi: 10.1186/s13063-023-07687-1 (PMC10694937; doi:10.1186/s13063-023-07687-1)
Supplement: Supplementary file 6 — Additional file 6. Data Monitoring Committee Membership. [file 13063_2023_7687_MOESM6_ESM.pdf]

Appendix 7 – Data Monitoring Committee Membership

NAME	AFFILIATION	EXPERTISE
Pollyanna Hardy (Chair)	National Perinatal Epidemiology Unit (NPEU)<br>Nuffield Department of Population Health<br>University of Oxford<br>UK	Clinical trialist, statistician and Director of the National Perinatal Epidemiology Unit (NPEU)
Olufunmilayo Fawole	Faculty of Public Heath<br>University of Ibadan<br>Nigeria	Professor of Epidemiology and Dean of the Faculty of Public Health, University of Ibadan
Andrew Weeks	International Maternal Health Care<br>University of Liverpool<br>Sanyu Research Unit<br>UK	Obstetrician. Professor of International Maternal Health.
