# Supplementary material for: Tranexamic acid by the intramuscular or intravenous route for the prevention of postpartum haemorrhage in women at increased risk: a randomised placebo-controlled trial (I’M WOMAN)
Source: Trials. 2023 Dec 3;24:782. doi: 10.1186/s13063-023-07687-1 (PMC10694937; doi:10.1186/s13063-023-07687-1)
Supplement: Supplementary file 7 — Additional file 7. Trial Steering Committee Membership. [file 13063_2023_7687_MOESM7_ESM.pdf]

Appendix 8 – Trial Steering Committee Membership

NAME	AFFILIATION	EXPERTISE
Jolly Beyeza-Kashesya (Chair)	Department of Obstetrics and Gynaecology, Mulago Specialised Women and Neonatal Hospital, Makarere University, Uganda	Senior Consultant, Obstetrics and Gynaecology
Rema Ramakrishnan	National Perinatal Epidemiology Unit, University of Oxford, UK	Senior Statistician; experienced biostatistician/epidemiologist
Ave Maria Semakafu	Institute of Development Studies, Muhimbili University of Health and Allied Sciences, Tanzania	Lecturer, patient representative/advocate
Saturday Etuk	University of Calabar, Nigeria	Chief Consultant & Professor of Obstetrics and Gynaecology
Aziz un-Nisa Abbasi	Department of International Medical College Abbottabad, Pakistan; Society of Obstetricians and Gynaecologists of Pakistan	Professor & Head of Obstetrics and Gynaecology; President of SOGON
Syeda Batool Mazhar	Shaheed Zulfiqar Ali Bhutto Medical University, Pakistan	Consultant & Professor of Obstetrics and Gynaecology
Nyanda Elias Ntinginya	NIMR Mbeya Medical Research Centre, Tanzania	Principal Research Scientist; Director; clinical trials expert
Ian Roberts	Clinical Trials Unit – Global Health Trials Group, London School of Hygiene & Tropical Medicine, UK	Chief Investigator; Professor of Epidemiology; clinical trials expert
Amy Brenner	Clinical Trials Unit – Global Health Trials Group, London School of Hygiene & Tropical Medicine, UK	Lead Investigator; clinical trials expert
