# Supplementary material for: Tranexamic acid by the intramuscular or intravenous route for the prevention of postpartum haemorrhage in women at increased risk: a randomised placebo-controlled trial (I’M WOMAN)
Source: Trials. 2023 Dec 3;24:782. doi: 10.1186/s13063-023-07687-1 (PMC10694937; doi:10.1186/s13063-023-07687-1)
Supplement: Supplementary file 8 — Additional file 8. List of Major Risk Factors for Postpartum Haemorrhage. [file 13063_2023_7687_MOESM8_ESM.pdf]

Appendix 9 – Major risk factors for postpartum haemorrhage

- increased maternal age (≥ 35 years)
- parity ≥ 4
- multiple pregnancy (2 or more foetuses)
- caesarean birth planned
- preterm birth (gestational age <37 weeks)
- previous PPH
- gestational hypertensive disorder of pregnancy (pre-eclampsia, eclampsia or hypertension)
- abnormal placental implantation (eg. placenta praevia, placenta accreta)
- antepartum haemorrhage or placenta abruption present
- moderate or severe anaemia (≤ 10 g/dL)
- foetal macrosomia
- intra-amniotic infection, e.g., prolonged rupture of membranes
- prolonged labour (>12 hours)
- fibroids
- uterine anomalies
- polyhydramnios
- dead foetus in utero
- obesity (BMI (body mass index) >35)
- gestational diabetes
